# BoT-Net: a lightweight bag of tricks-based neural network for efficient LncRNA–miRNA interaction prediction

**DOI:** 10.1007/s12539-022-00535-x

**Published:** 2022-08-10

**Authors:** Muhammad Nabeel Asim, Muhammad Ali Ibrahim, Christoph Zehe, Johan Trygg, Andreas Dengel, Sheraz Ahmed

**Affiliations:** 1 Department of Computer Science, Technical University of Kaiserslautern, 67663 Kaiserslautern, Rhineland-Palatinate Germany; 2 German Research Center for Artificial Intelligence GmbH, 67663 Kaiserslautern, Rhineland-Palatinate Germany; 3 Sartorius Stedim Cellca GmbH, 88471 Laupheim, Baden-Wurttemberg Germany; 4 Computational Life Science Cluster (CLiC), Umea University, 90187 Umea, Sweden

**Keywords:** Deep learning, Long non-coding RNA, Micro-RNA, Bag of tricks, Deep learning strategies, Robust interaction predictor, lncRNA–miRNA interaction prediction, Lightweight neural network

## Abstract

****Background and objective**:**

Interactions of long non-coding ribonucleic acids (lncRNAs) with micro-ribonucleic acids (miRNAs) play an essential role in gene regulation, cellular metabolic, and pathological processes. Existing purely sequence based computational approaches lack robustness and efficiency mainly due to the high length variability of lncRNA sequences. Hence, the prime focus of the current study is to find optimal length trade-offs between highly flexible length lncRNA sequences.

****Method**:**

The paper at hand performs in-depth exploration of diverse copy padding, sequence truncation approaches, and presents a novel idea of utilizing only subregions of lncRNA sequences to generate fixed-length lncRNA sequences. Furthermore, it presents a novel bag of tricks-based deep learning approach “Bot-Net” which leverages a single layer long-short-term memory network regularized through DropConnect to capture higher order residue dependencies, pooling to retain most salient features, normalization to prevent exploding and vanishing gradient issues, learning rate decay, and dropout to regularize precise neural network for lncRNA–miRNA interaction prediction.

****Results**:**

BoT-Net outperforms the state-of-the-art lncRNA–miRNA interaction prediction approach by 2%, 8%, and 4% in terms of accuracy, specificity, and matthews correlation coefficient. Furthermore, a case study analysis indicates that BoT-Net also outperforms state-of-the-art lncRNA–protein interaction predictor on a benchmark dataset by accuracy of 10%, sensitivity of 19%, specificity of 6%, precision of 14%, and matthews correlation coefficient of 26%.

****Conclusion**:**

In the benchmark lncRNA–miRNA interaction prediction dataset, the length of the lncRNA sequence varies from 213 residues to 22,743 residues and in the benchmark lncRNA–protein interaction prediction dataset, lncRNA sequences vary from 15 residues to 1504 residues. For such highly flexible length sequences, fixed length generation using copy padding introduces a significant level of bias which makes a large number of lncRNA sequences very much identical to each other and eventually derail classifier generalizeability. Empirical evaluation reveals that within 50 residues of only the starting region of long lncRNA sequences, a highly informative distribution for lncRNA–miRNA interaction prediction is contained, a crucial finding exploited by the proposed BoT-Net approach to optimize the lncRNA fixed length generation process.

****Availability**:**

BoT-Net web server can be accessed at https://sds_genetic_analysis.opendfki.de/lncmiRNA/.

**Graphic Abstract:**

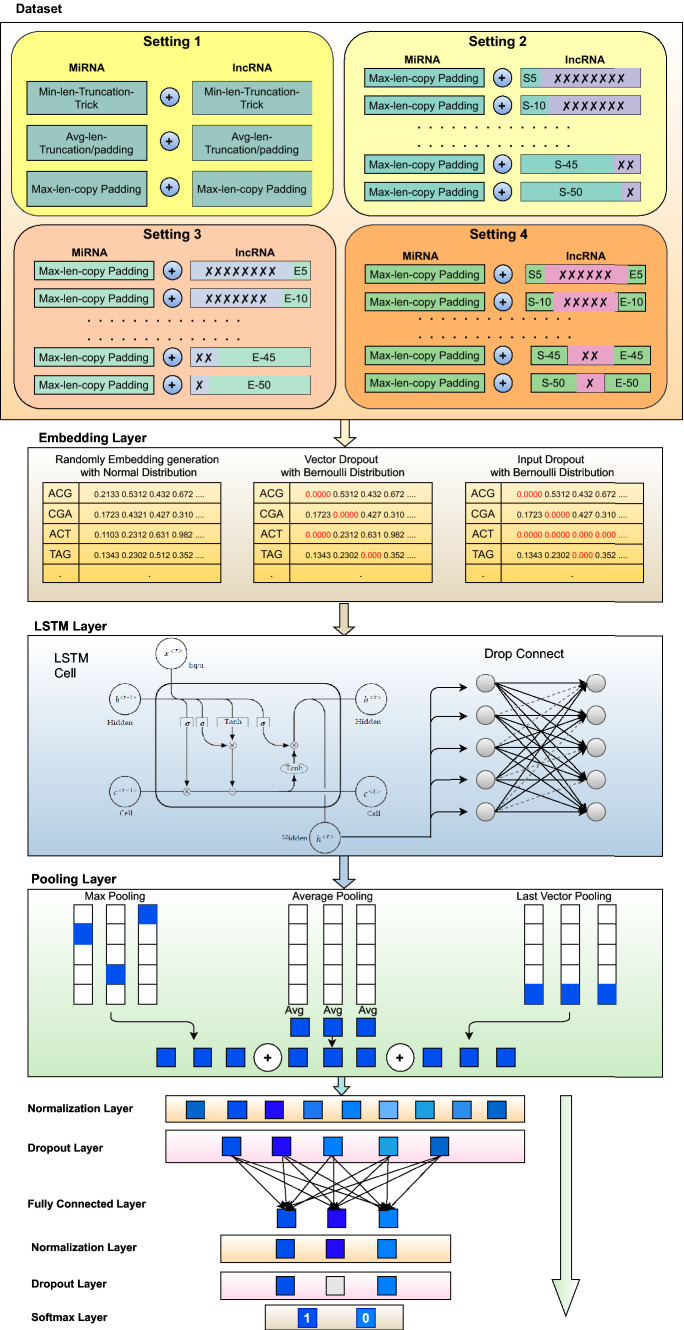

## Introduction

Understanding physiological and biological functionalities of known non-coding ribonucleic acids (ncRNAs) [44, 61] and discovering new types of ncRNAs are important fields of interest in genetic research [22, 57]. Long ncRNAs (lncRNAs) and micro-RNA (miRNAs) are sub-classes of ncRNAs which are found to play an important role in a variety of biological processes [72]. MiRNAs contain 17 to 25 residues and contribute significantly to post-transcriptional regulation of gene expression [81, 95]. Unlike miRNAs, lncRNAs sequence lengths largely vary from 200 to 23,000 residues [30]. LncRNAs are involved in epigenetic, transcriptional, post-transcriptional regulation of gene expression [83, 98], chromatin remodeling [39, 64], as well as activation or repression of immune responses [42]. Despite their differences, both ncRNAs have strong relations with each other and are responsible to direct many biological processes [72, 86].

LncRNAs and miRNAs play significant roles in cell differentiation and proliferation [63]. Furthermore, growing evidences show that the interaction between lncRNAs and miRNAs plays an imminent role in gene regulation [77], cellular metabolic processes [77], development of cardiovascular diseases, Alzheimer, Liver fibrosis, and Cancer [5, 38]. LncRNAs can act as sponges to control the functionality of miRNAs, whereas miRNAs can act as decoys and trigger the decay of lncRNAs [67]. Identification of lncRNA–miRNA interactions is essential to understand the function of lncRNAs, miRNAs, and their role in biological and pathological processes [29, 67]. A detailed understanding of lncRNA–miRNA interactions paves way for biomarker discovery and the development of therapeutics.

Although experimental methods such as affinity purification [32], ChIP-PCR [89], and Double-Luciferase Reporter assays [89] are successfully used to detect lncRNA–miRNA interactions. They tend to be costly, labour intensive, time-consuming and prone to errors [82]. With the tremendous success of deep neural networks in Computer Vision [69], Natural Language Processing [56], and Bioinformatics [88], a number of computational methodologies have been presented to infer the interaction between lncRNAs and miRNAs. These approaches utilize either known intrinsic information of lncRNA and miRNA sequences such as expression profile similarity-based network, functionality similarity-based network or raw sequence information. For example, considering that lncRNAs and miRNAs with similar expression profiles are more likely to interact, Huang et al [25] developed a group-preference Bayesian collaborative filtering (GBCF) predictor for predicting the interaction between lncRNAs and miRNAs. GBCF evaluation was performed using biological functionality-based lncRNA–miRNA similarity, expression profile-based similarity, and sequence similarity-based features. Empirical evaluation indicated that GBCF achieved better performance using expression profile-based similarity features followed by biological functionality-based similarity features. Huang et al [23] developed an expression profile-based classifier (EPLMI) that leveraged gene-based miRNA similarity and functional relatedness of lncRNA to infer potential lncRNA–miRNA interactions.

Huang et al. [26] presented a graph-convolution auto-encoder-based deep learning model namely “GCLMI” that used expression profiles of lncRNAs and miRNAs. Using expression profiles of lncRNAs and miRNAs, Wang et al. [75] developed a deep learning model LMI-DForest that first learned the latent space of miRNA and lncRNA sequences. Then, compressed latent space representation was passed to a deep forest network to predict potential lncRNA–miRNA interactions. Zhao et. al [99] presented LMMAN built upon molecular association graphs to infer interactions among LncRNAs–miRNAs. To construct molecular association graphs, known relationships between lncRNAs, miRNAs, diseases, and drugs were integrated together. Afterwards, a large-scale information network embedding (LINE) approach was utilized to acquire network behaviour related features of miRNA and lncRNA nodes. Finally, a random forest predictor was used to infer potential interactions among miRNA and lncRNA sequences. Similarly, there exist several other lncRNA–miRNA interaction prediction approaches that leverage known intrinsic information of lncRNA and miRNA sequences to determine lncRNA–miRNA interaction in various species [6, 14, 19, 20, 43, 43, 74, 78, 90, 91, 93, 96, 97, 100–103].

On the other hand, computational approaches that solely utilize sequence information, have also been proposed for lncRNA–miRNA interaction prediction task. Kang et al. [33] fed RNA sequences and 110 sequence-specific features to a hybrid model named as PmliPred. PmliPred utilized a random forest and bi-directional gated recurrent unit for the inference of lncRNA–miRNA interactions. However, the bottleneck issue of PmliPred is the manual extraction of sequence-related features that require extensive time and expert knowledge. In addition to substantial manual effort, it is also likely that the selected features may not perform well for the identification of interactions between biomolecules of different species [58]. Zhou et al. [104] presented an ensemble-graph embedding approach (GEEL) that leveraged known interactions and the linear neighborhood similarity technique to generate a lncRNA–miRNA interaction network. Using this network, they developed 4 graph embeddings namely Laplacian Eigenmaps, GraRep, DeepWalk, High-order closeness reserved embeddings, and a graph-based auto-encoder to learn a rich statistical representation of LncRNA and miRNA sequences represented as nodes in the graph. Using developed embeddings and random forest estimator, GEEL predicted the interactions between miRNAs and lncRNAs. Recently, Yang et al. [82] presented another sequence information-based predictor LncMirNet. LncMirNet combined physico-chemical property-based encoding learned through composition transition distribution with residue frequency-based encoding learned using doc2vec embedding generation approach. To generate an aggregated matrix for differently learned sequence features, histogram-dd approach was employed. Finally, the constructed matrix was passed to a convolutional neural network (CNN) which inferred the potential LncRNAs–miRNAs interactions.

Critical analysis of existing computational approaches indicates that for expression profiles similarity-based lncRNA–miRNA interaction prediction approaches [23, 25], a tremendous amount of expression profiles of diverse human tissues and cell lines need to be collected to achieve decent predictive performance [82]. Although data related to the biological profiles of lncRNA and miRNA are consistently accumulated, however, considering the dynamic nature of regulatory mechanisms of lncRNAs and miRNAs, it is not feasible to develop a complete lncRNA–miRNA interaction network. This is why expression profile similarity-based approaches are not applicable to novel lncRNA–miRNA pairs, expression profiles of which may not have any links within the known lncRNA–miRNA interaction network [23]. Furthermore, such approaches are not adaptable for a large community of researchers as the collection of comprehensive biological information requires extensive time and controlled environments. On the other hand, graph-based lncRNA–miRNA interaction prediction approaches lack in ability to handle intrinsic features of miRNAs and lncRNAs, including sequence and structural information.

In contrast, purely sequence information-based computational lncRNA–miRNA interaction prediction approaches are more promising as they are scalable and widely adaptable. The main focus of existing sequence information-based computational predictors has been to effectively encode the relationships that exist between the residues of lncRNA and miRNA sequences. However, existing computational approaches show limited performance and a lack of robustness primarily due to the huge fluctuation in the length of lncRNA sequences. Just like usual deep learning approaches, state-of-the-art method LncMirNet [82] operates on fixed-size lncRNA–miRNA sequence pairs. Unlike miRNA sequences, lncRNA sequences are of highly variable lengths and to generate a homogeneous representation of such sequences, fluctuation in sequence length is handled by truncating the sequences to the minimum/average length (sequence truncation trick) or mapping them to maximum sequence length (copy padding trick). Considering the extreme length variability of lncRNA sequences, sequence truncation to the minimum or average length loses important information related to discriminative residue distribution. However, copy padding trick introduces a substantial bias through the addition of too many zeros especially in low-dimensional sequences which eventually makes a large number of lncRNA sequences very much identical to each other. This phenomenon largely deteriorates the classifier [82] ability to distinguish various lncRNAs which eventually derail lncRNA–miRNA interaction prediction performance.

Instead of feeding entire lncRNA sequences to a deep learning model [82, 104], here we investigate which region of lncRNA sequences (e.g starting region, ending region, starting–ending region) contains the most informative residue distribution for interaction prediction. A rigorous experimentation with benchmark dataset is performed to find and retain sub-sequences that can precisely capture the biological essence of very flexible and long lncRNA sequences. Optimal lncRNA sub-sequences are combined with complete miRNA sequences and passed to a newly developed bag of tricks-based neural network (BoT-Net). BoT-Net does not rely on expensive knowledge graphs, expression profiles, regulatory functionalities, or sequence similarity metrics to better understand the correlations of lncRNA–miRNA sequences. BoT-Net leverages a single layer long short-term memory network regularized through DropConnect to effectively capture long-range dependencies of higher order residues, three kinds of pooling strategies to retain most discriminative features, normalization to prevent exploding and vanishing gradient issues, learning rate decay, and dropout to avoid over-fitting. Using only sequence information, smart integration of multiple extremely effective deep learning tricks [17, 28, 31] help BoT-Net capture local and global dependencies of higher order residues, converge faster, and reduce the generalizability error for lncRNA–miRNA interaction prediction.

Rich performance comparison of traditional copy padding, sequence truncation, and sub-sequence generation approaches is performed using the benchmark dataset to indicate the discriminative potential of different regions of lncRNA sequences for lncRNA–miRNA interaction prediction. Furthermore, a detailed performance comparison of the proposed BoT-Net with existing sequence information-based computational lncRNA–miRNA interaction predictors over a benchmark dataset indicates that BoT-Net methodology outperforms the state-of-the-art approach by 2%, 4%, and 8% in terms of accuracy, matthews correlation coefficient, and specificity solely using few residues from the starting region of long lncRNA sequences. Furthermore, a case study analysis indicates that BoT-Net methodology manages to retain promising performance trends on a different benchmark dataset namely RP1369 [105] for the task of lncRNA–protein interaction prediction, achieving the top accuracy of 73% and outperforming state-of-the-art lncRNA–protein interaction predictor [105] by 10% using few residues from only starting region of lncRNA and protein sequences. Considering the unique design of BoT-Net methodology, we anticipate that the paradigm of keeping only the most informative residue distribution-based sub-sequences will open new horizons for interaction inference research related to other non-coding RNAs.

## Methods

This section illustrates the proposed lncRNA–miRNA interaction prediction methodology (BoT-Net), benchmark dataset, and evaluation metrics used to evaluate the integrity of BoT-Net.

### BoT-Net: a bag of tricks-based neural network for efficient lncRNA–miRNA interaction prediction

For the interaction prediction problem, there exist two paradigms for the development of a deep learning-based end-to-end predictor. One paradigm promotes the development of a multi-head neural network in which miRNA sequences are passed at one head and lncRNA sequences are passed at the second head [73]. Both heads extract features from different sets of sequences which are concatenated before passing to the final classification layer [73]. Whereas, the second paradigm combines lncRNA and miRNA sequences to formulate lncRNA–miRNA sequence pairs where every pair is treated as a single instance. These pairs are passed to a single head neural network which extracts important features before passing forward to the final classification layer [35]. In the proposed BoT-Net methodology, we use a single-head neural network to distinguish interactive lncRNA–miRNA pairs from non-interactive lncRNA–miRNA pairs.

**Fig. 1:**
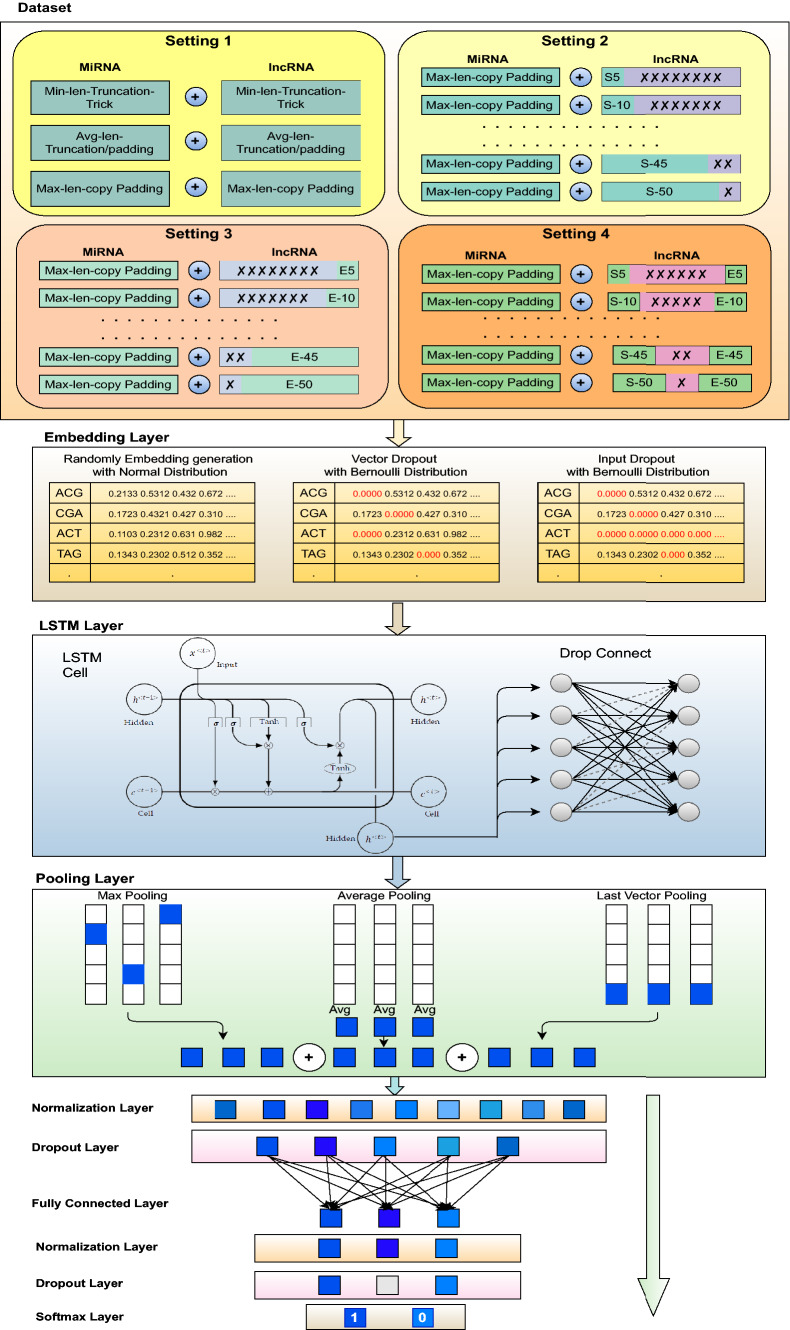
Workflow of proposed lightweight bag of tricks based neural network (BoT-Net) for lncRNA–miRNA interaction prediction

Workflow of proposed lncRNA–miRNA interaction prediction methodology “BoT-Net” can be segregated into three different modules. First, it generates fixed-length sequences using traditional copy padding or sequence truncation tricks as well as sub-sequences based on the highly informative and discriminative distribution of residues. Paradigms of four different fixed-length sequence generation approaches are illustrated at top of the Fig. 1, a precise description of which is given in the Sect. 2.1.1. Second, it generates higher order residues of lncRNA–miRNA sequences, whose statistical representation is learned using randomly initialized neural embeddings based on normal distribution where the embedding matrix is optimized using two different kinds of dropout strategies. Precise workflow of mapping higher-order residues into vector space is depicted in the middle of the Fig. 1, details of which are given in Sect. 2.1.2. Using the statistical representation of most informative lncRNA sub-sequence-based fixed-length lncRNA–miRNA pairs, in the final module, a bag of tricks-based precise neural network is trained. The workflow of final module is demonstrated at bottom of the Fig. 1, detailed description of classifier and optimization components is given in Sect. 2.1.3 and following sub-sections.

#### Fixed length generation of lncRNA–miRNA sequences

Deep learning approaches require a fixed-length representation of genomic sequences to model the complex relationships between residues. Pre-dominantly, for multifarious genomics and proteomics sequence analysis tasks, variable length sequences are transformed into fixed-length sequences using a copy padding trick. For instance, Zhang et al. [94] revealed the impact of copy padding trick on the generalizability of convolutional neural network for lncRNA–protein interaction prediction. Likewise, Lopez et al. [45] explored the impact of copy padding trick for archaeal protein function prediction task. However, existing studies do not explore the impact of various other sequence fixed-length generation approaches such as sequence truncation approach or hybrid approach which combines copy padding with sequence truncation approach. The paper in hand performs comprehensive experimentation with three different pre-processing strategies for lncRNA–miRNA interaction prediction including copy padding, sequence truncation, and hybrid approach, shown under the hood of setting-1 in Fig. 1.

In copy padding trick, first, corpus sequences lengths are compared to find a maximum sequence length. Then, a certain constant is added in those sequences whose length is less than the maximum length to justify all sequence lengths with respect to the maximum length. Sequence truncation is another sequence fixed-length generation approach that relies on the minimum possible sequence length. Residues from all those sequences are truncated whose length is greater than the minimum length to justify all sequence lengths with respect to the minimum length. Another trend is to use a hybrid approach that makes use of both copy padding and sequence truncation tricks by computing average sequence length. In this case, a particular constant is added in those sequences whose sequence length is less than the average length, whereas sequences longer than average length are trimmed to justify all sequence lengths with respect to the average length.

In copy padding trick, it is an important matter to determine whether the start of the sequences is more suitable for the addition of a particular constant or end of the sequences. Similarly, in the sequence truncation trick, it is debatable whether extra residues need to be removed from the end of the sequences or start of the sequences. Pre-dominantly, end of the sequences has been chosen for extension or truncation [7, 10, 36, 50, 71, 85, 94], therefore, in the 1$^\text {st}$ setting, the paper in hand performs experimentation by extending or trimming the sequences from the end. To find which trick performs better among all trivial copy padding or sequence truncation tricks, it performs a detailed performance comparison by taking all three traditional sequence fixed-length generation paradigms into account. A graphical illustration of all three fixed sequence length generation strategies is given in Fig. 1 under the umbrella of setting-1.

In existing lncRNA–miRNA interaction prediction approaches, variable length lncRNA–miRNA sequences are transformed into fixed-length sequences through traditional copy padding or sequence truncation tricks [33, 82, 104]. In benchmark lncRNA–miRNA interaction prediction dataset, miRNA sequence length varies from 17-to-25 residues where average sequence length falls around 22 residues. Taking into account the light length variability of miRNA sequences, following the most common trend in the literature [7, 10, 33, 36, 50, 71, 82, 85, 94, 104], we utilize the copy padding trick to map all miRNA sequences to maximum length ( 25 residues).

Unlike miRNA sequences, length of lncRNA sequences largely fluctuate. In the benchmark lncRNA–miRNA interaction prediction dataset, lncRNA sequence length varies from 213-to-22,743 residues. As lncRNA sequences are highly flexible in length which is why fixing the length of lncRNA sequences is a quite difficult task. Although the copy padding trick effectively handles the light length variability of miRNA sequences, however, it does not work well for lncRNA sequences. This is primarily because lncRNA average sequence length falls around 1424 residues and copy padding trick introduces a significant level of bias through the addition of too many zeros which make a large number of lncRNA sequences very similar to each other. For lncRNA sequences, copy padding trick derails the classifier ability to distinguish different lncRNA sequences which badly impacts overall interaction prediction performance.

To optimize trivial sequence fixed-length generation paradigm by effectively handling the high length variability of lncRNA sequences, this paper proposes a novel idea that finds and retains only the most informative lncRNA sub-sequences. The paper in hand performs experimentation with three distinct settings to assess whether sub-sequences taken from diverse regions of lncRNA sequences contain a highly informative and discriminative distribution of residues and are capable to surpass the predictive potential of traditional copy padding or sequence truncation tricks for the task of lncRNA–miRNA interaction prediction.

We consider only a few residues from the starting region of the lncRNA sequences, ending region of lncRNA sequences, or the start-end region of lncRNA sequences. Figure 1 depicts the most informative and discriminative residue distribution-based settings presented to generate optimal fixed length lncRNA sub-sequences that are entitled as setting-2, setting-3, and setting-4 respectively. In presented settings, we consider a small number of residues like 5 which is approximately 3% of the minimum lncRNA sequence length of the benchmark dataset and continue to increase this number to 25% of the average lncRNA sequence length using a certain step size. In particular, in the 2$^\text {nd}$ setting, BoT-Net selects X residues merely from the starting region of lncRNA sequences. In the 3$^\text {rd}$ setting, BoT-Net selects Y residues only from the ending region of lncRNA sequences. In 4$^\text {th}$ setting, BoT-Net combines X residues taken from the start of lncRNA sequences with Y residues taken from the end of lncRNA sequences to assess the discriminative potential of the start-end region. In all three settings, values of X and Y differ from 5-to-50 taken with the step size of 5. Evaluating the predictive potential of diverse regions of lncRNA sequences assists to identify whether precise lncRNA sub-sequences manage to obtain discriminative essence of lncRNA sequences which improves generalizability of the classifier.

Fusing miRNA sequences with lncRNA sequences, fixed-size sequences generated using diverse pre-processing strategies are passed forward to higher order residue generation and statistical representation learning module.

#### Higher order residue embedding generation and optimization

**Fig. 2:**
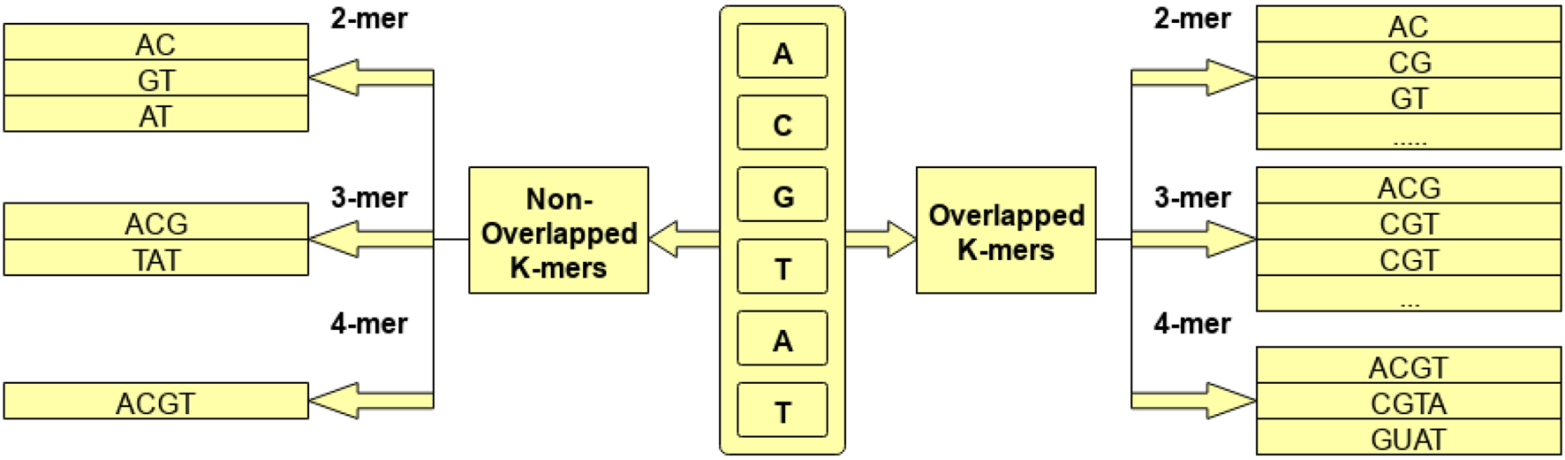
Process of generating different overlapping and non-overlapping higher order residues

To create any kind of statistical representation of sequences, first step is to generate higher order residues of sequences. Higher order residues can be generated in two different manners, one way is to generate overlapping higher order residues in which a fixed size window is rotated over the sequences with the stride size smaller than the size of the window. For instance, if we have to generate second-order residues of the sequences, we will rotate a window of size 2 with the stride size of 1. In the second approach, non-overlapping higher-order residues are generated in which a fixed size window is rotated over sequences with stride size equal to the size of the window. Figure 2 describes the process of generating overlapping and non-overlapping higher order residues. As lncRNA–miRNA sequences are comprised of four basic residues Adenine (A), Cytosine (C), Guanine (G), and Uracil (U), therefore, the size of the vocabulary can be computed using $4^k$. The value of *k* eventually determines the size of vocabulary which impacts memory cost, runtime cost, model generalizability, and up to what extent residue order and semantic properties are taken into account. Therefore, the choice of *k* is a very crucial task in achieving better predictive performance. Considering the work of Asim et al. [2] and Le et al. [1], this paper utilize the stride size of to generate overlapping higher order residues (5-mers).

**Statistical Representation Learning**

To generate the statistical representation of higher order residues present in lncRNA–miRNA sequences required by deep learning models, in literature, physico-chemical property-based encoding, pre-trained higher order embeddings, and one-hot encoding are commonly applied [33, 82, 104]. Learning higher order residue representation through physico-chemical properties requires evaluation of a tremendous amount of properties having unique characteristics [8, 54]. Also, physico-chemical property-based encoding lacks to capture global dependencies of residues present in genomic sequences. One-hot encoding also proves in-efficient as it does not take positional information of residues into account [11, 12, 53, 65, 66, 80]. Whereas, pre-trained neural higher order residue embeddings require rigorous training over large corpora (e.g Swiss-Prot) and fine-tuning to adapt the local context and prove effective for downstream classification tasks.

Following the work of Elabd et al. [13] and Asim et al. [3], the paper in hand proposes the idea of learning higher-order residue neural embeddings by randomly initializing the embedding matrix using the Pytorch embedding layer for the task of lncRNA–miRNA interaction prediction.

The embedded layer generates a two-dimensional weight matrix or lookup table $E \in \mathbb {R}^\text {vocab x n}\_\text {embedding}$ where each row indicates a vector representation of sequence residue. While the number of rows is equal to the unique residues present in the vocabulary, the number of columns indicates the embedding dimensions $n\_embedding$. In our experimentation, initially, the embedding matrix is randomly initialized where every higher-order residue is represented as a 120-dimensional vector using the normal distribution. However, during training, embedding matrix is updated to minimize the predictive error. Optimized embeddings for each higher-order residue are learned through an iterative process of updating the weights of the embedding matrix. This approach largely differs from classical one-hot encoding, physico-chemical property-based encoding, and higher order residue frequency-based encoding schemes where the numerical value of higher order reside is not updated during the training.

To further optimize the embedding matrix, we apply two different kinds of dropout schemes where each higher order residue vector has the probability $p_{embeddings}$ of 0.004 to be dropped and every vector weight unit has the likelihood of 0.005 to be dropped [16, 51]. Embedding and weight unit dropout schemes assist to capture rich interactions with light memory and run time cost. Optimized higher order residue embedding-based statistical representation of lncRNA–miRNA sequences is passed to the BoT-Net interaction prediction classifier.

#### BoT-Net classifier

The complete workflow of the proposed bag of tricks-based neural network BoT-Net is depicted in Fig. 1. For each fixed-length lncRNA–miRNA sequence, overlapping higher order residues are generated, representation of which is learned using randomly initialized embeddings. After mapping higher order residues into vector space, 120-dimensional statistical representation of lncRNA–miRNA sequences is passed to a single layer LSTM regularized using DropConnect approach. The output of LSTM is passed to the pooling layer which makes use of 3 different strategies to generate 360-dimensional sequence vectors having highly informative features. Pooled features are then passed to the batch normalization layer that normalizes the given distribution within a mean of zero and variance of one to alleviate the internal co-variance shift. To avoid over-fitting, large weights of the neural network are penalized using learning rate decay. Before feeding normalized features to a fully connected layer, standard dropout is applied. A fully connected layer transforms 360-dimensional sequence vectors into 50-dimensional sequence vectors which are once again passed through batch normalization and dropout layers before passing to the softmax classification layer. Different phases of the proposed BoT-Net approach are briefly discussed in the following subsections.

**Lightweight long short-term memory (LSTM) network**

Proposed BoT-Net methodology leverages a single layer LSTM which unlike traditional recurrent neural network (RNN) is far more capable of retaining long-term dependencies using update gate. Furthermore, LSTM filters information at every time step and is far less vulnerable to vanishing gradients issue. Mathematically, it can be expressed as follows:

1 $$\begin{aligned} i_{t}&= \sigma (W^{i}.x_{t}+U^{i}.h_{t-1}) \end{aligned}$$

2 $$\begin{aligned} f_{t}&= \sigma (W^{f}.x_{t}+U^{f}.h_{t-1}) \end{aligned}$$

3 $$\begin{aligned} o_{t}&= \sigma (W^{o}.x_{t}+U^{o}.h_{t-1}) \end{aligned}$$

4 $$\begin{aligned} cin_{t}&= tanh (W^{c}.x_{t}+U^{c}.h_{t-1}) \end{aligned}$$

5 $$\begin{aligned} c_{t}&= (i_{t} \odot cin_{t}+ f_{t} \odot +cin_{t-1} \end{aligned}$$

6 $$\begin{aligned} h_{t}&= (o_{t} \odot tanh c_{t}) \end{aligned}$$

Here, [$W^{i}$, $W^{f}$, $W^{o}$, $U^{i}$, $U^{f}$, $U^{o}$] refer to weight matrices, $x_{t}$ represents the 120-dimensional higher order residue vector fed at at time-step t, $h_{t}$ refers to current hidden state, $c_{t}$ is the state of memory cell, and $\odot$ represents the element wise product.

To avoid over-fitting recurrent neural networks (RNNs), researchers have applied diverse regularization techniques mostly on hidden state vector $h_{t-1}$. More specifically, applying standard dropout between time steps or upon updating the memory state $c_{t}$ is quite common where randomly chosen activations are turned to zero. However, in this study, we utilize a generalization of dropout called DropConnect [70] with probability of 0.004. Unlike standard dropout, DropConnect applies dropout operation on hidden-to-hidden weight matrices of LSTM before forward and backward pass. As the weights are reused over many time steps, dropped weights will not make any contribution across the entire forward or backward pass. Aggregated gradients of each 64 lncRNA–miRNA sequence batch are back-propagated through time without resetting the hidden state of LSTM.

**Vector Pooling**

The prime goal of applying vector pooling is to acquire highly representative features of sequences. Consider the example of disease classification where those features will be the most informative features that describe health-related complications. Selecting an effective pooling strategy from a variety of strategies is extremely crucial to effectively acquire the essence of sequences which aids to achieve optimal predictive performance. In order to effectively handle the location-invariance of different features extracted by LSTM layer and to retain the most salient features by eliminating redundant features, 3 different kinds of pooling operations are applied. More specifically, we use maximum, average pooling, and last vector pooling. Maximum pooling aims to retain the most discriminative features and discard less imminent features by rotating a fixed-sized 1-d window (Eq. 7).

7 $$\begin{aligned} po_k^j=max\{c^j_{k:k+r-1}\} \end{aligned}$$

Average pooling computes the mean of values for each pooling region to reveal that, up to what extent (on average) a lncRNA–miRNA pair is interactive or non-interactive (Eq. 8).

8 $$\begin{aligned} av_k^j=average\{c^j_{k:k+r-1}\} \end{aligned}$$

In these equations, $$po_k^j$$$$c_j$$ refers to the input, r represents the pool size, denotes the maxim value is respective pooling block, and $$av_k^j$$ denotes the average value corresponding to the pooling block. Vectors resulting from maximum and mean pooling operations are combined with the last higher order residue 120-dimensional vector to generate a 360-dimensional vector for each lncRNA–miRNA sequence which is passed further in the network.

**Batch Normalization**

Normalizing the input distribution in such a way that it fulfills the mean criteria of zero and constant standard deviation [40] proves beneficial for the training of neural networks. Deep neural networks face the issue of internal co-variance shift where the distribution of intermediate layers of neural networks significantly fluctuates due to the change in input distribution [27]. Internal co-variance shift makes the weights of neural network learned during previous iterations totally obsolete [27]. Internal co-variance de-stabilizes model convergence and deteriorate the generalizability of the model [27]. To handle the problem of internal co-variance shift, batch normalization extends the paradigm of input normalization across the hidden layers within a deep neural network, where the normalization is performed over the mini-batches for speed reasons [27]. Batch normalization has achieved great success in multifarious areas of deep learning [4, 21, 60] as it makes sure that input to output mapping of the deep neural network does not over-specialize only a particular block of input distribution which results in faster training, better convergence, and generalizability [27]. Mathematically, providing the d-dimensional feature space $x=\{x^{(1)},........,x^{(d)}\}$, batch normalization operation can be expressed as follows:

9 $$\begin{aligned} \tilde{x^{(k)}}=\frac{x^{(k)} - E[x^{(k)}]}{\sqrt{Var [x^{(k)}]}}, h^{(k)}=\gamma ^{(k)} \tilde{x^{(k)}} + \beta ^{(k)} \end{aligned}$$

Here, $x^{(k)}$ denotes the k-th activation of the input and $h^{(k)}$ denotes k-th activation of the output, E[.] and Var[.] represent the expectation and variance whereas $\gamma ^{(k)}$ and $\beta ^{(k)}$ are the hyperparameters learned during an iterative training phase.

**Standard Dropout**

To reduce noise and assist the softmax classification layer in distinguishing interactive and non-interactive lncRNA–miRNA pairs, we apply the standard dropout [62] on pooled normalized features. It is widely considered a de-facto standard way of regularizing deep neural networks which face the issue of over-fitting. Standard dropout randomly drops units during every iteration of gradient descent and forces the network to learn multiple mappings from input to output. However, an ineffective application of dropout works poorly and can even further increase the generalization error in RNNs [51]. Like dropping unit at each time step significantly disrupts network capability to retain long-term dependencies [16]. Therefore, one needs to apply dropout quite smartly. In the proposed BoT-Net methodology, each unit has the likelihood of 0.1 to be dropped. Mathematically (Eq. 10), probability of dropping a unit is done following the Bernoulli distribution using a probability p. Taking the dot product of a unit vector with the mask where every element is sampled randomly using Bernoulli distribution, units are dropped during the training phase. On the other hand, for the testing phase (Eq. 11), rather than dropping the units, the likelihood for a unit not to be dropped $1-p\%$ is computed.

10 $$\begin{aligned} y&=f(Wx)\bullet m, m_i\sim Bernoulli(p) \end{aligned}$$

11 $$\begin{aligned} y&=(1-p)f(Wx) \end{aligned}$$

**Weight Decay**

Weight decay is another regularization strategy that primarily penalizes the large weights of the model [47, 87]. Generally, the weight decay strategy can be applied in two different ways. The first approach is called L2 regularization where the sum of the squared weight is directly added in the loss value, which can be mathematically represented as:

12 $$\begin{aligned} L_{\text{ t }otal} = \Big (L + \lambda ||w||_2^2\Big ) \end{aligned}$$

On the other hand, in the second technique, weight is added during the update of gradients, which mathematically can be expressed as:

13 $$\begin{aligned} w_{i+1} = w_i - 2 \lambda w_i - \Big <\frac{\delta L}{\delta w }\vert _{w_i}\Big >\end{aligned}$$

For simple optimizers like stochastic gradient descent, both weight decay strategies work in the same manner. But for more sophisticated optimizers such as Adam who accumulates the gradients, weight decay and L2 regularization impact greatly differ from one another. For the L2 regularization, the sum of squared weights is added to the loss value, making it an integral part of the gradient. Thereby, when the Adam optimizer accumulates the gradients, L2 penalty terms are accumulated as well.

In contrast, in the second approach, weight decay is only added during the update step, indicating that the accumulation of weight terms during the gradient is not performed. Unlike the L2 regularization strategy, the weight decay strategy has shown more improvement in the performance [46], therefore proposed BoT-Net is trained using the batch size of 64 with weight decay strategy where the value of learning rate is 0.01, value of weight decay is 0.02, and ADAMW is used as an optimizer.

**Fully Connected Layer**

A fully connected layer examines 360-dimensional pooled features, alleviates dimensionality, and learns a compact 50-dimensional representation of lncRNA–miRNA sequences. Learned compact representation is passed to another batch normalization followed by dropout layer before feeding to softmax classification layer which predicts the probability for interactive and non-interactive classes. Mathematically, calculations of a fully connected layer are equivalent to trivial perception which can be expressed as:

14 $$\begin{aligned} fc^l=\sigma (w^l x fc^l + b^l ) \end{aligned}$$

Here, $fc^l$ denotes the output features produced by the l-th full connected layer, $w^l$ and $b^l$ represent the weight and bias, while $\sigma (.)$ refers to the non-linear activation function used in the experimentation.

**Softmax**

Softmax classification layer discriminates interactive lncRNA–miRNA pairs from non-interactive lncRNA–miRNA pairs. Categorical cross-entropy loss is the training objective which is a simple softmax activation plus a cross-entropy loss. Working of softmax (Eq. 15) and categorical cross-entropy (Eq. 16) can be represented using following mathematical expressions:

15 $$\begin{aligned} f(s_i)&=\frac{e^s_i}{\sum _{j}^{C} e^s_j} \end{aligned}$$

16 $$\begin{aligned} CE&=-\sum _{i}^{C} t_i log(f(s_i)) \end{aligned}$$

Here, *t* denote one-hot encoded actual class label, $s_i$ refers to probability score estimated for every class present in C and $f(s_i)$ represents softmax activation applied before the estimation of cross-entropy loss.

### Benchmark datasets

To evaluate the integrity of proposed BoT-Net methodology and to perform a fair comparison with state-of-the-art LncRNA–miRNA interaction predictor [82], we perform experimentation on a benchmark dataset provided by Yang et al. [82].

To compile true LncRNAs–miRNAs interaction corpus, first LncRNAs and miRNA IDs were extracted from lncRNASNP2 database by Yang et al. [82]. More specifically, only those lncRNA–miRNA pairs were selected where the corresponding record in lncRNASNP2 database was shown “ENST” and “hsa-miR” simultaneously. Afterwards, using extracted IDs of both non-coding RNAs, LncRNA sequences of homo sapiens were acquired from GENCODE^1^[15] and miRNA sequences were obtained from miRbase metathesaurus^2^[37]. In this manner, a total of 1663 lncRNAs, 258 miRNAs, and 15,386 true lncRNA–miRNA sequence pairs along with interaction information were obtained.

Authors utilized random pairing strategy to generate non-interactive samples of lncRNA and miRNA sequences. More specifically, after shuffling lncRNA and miRNA sequences 10 times using Knuth-Durstenfeld shuffling paradigm [48], iteratively, an arbitrarily selected lncRNA–miRNA pair was placed in the negative set, provided the same pair was not present in the positive set which contains interactive lncRNA–miRNA sequence pairs. In this manner, 15,386 negative lncRNA–miRNA sequence pairs were generated.

Furthermore, to evaluate the generalizeability of proposed BoT-Net methodology, we perform experimentation on another benchmark dataset namely RP1369 [105] for the task of lncRNA–protein interaction prediction. Shin et al. [105] developed the RP1369 dataset by collecting 943 RNA-protein complexes from protein–RNA interface database (PRIDB) [106]. The RP1369 dataset contained a total of 369 positive lncRNA–protein interactions which involve 331 lncRNA and 338 proteins. Authors extracted 369 non-interactive lncRNA protein sequence pairs from the datasets used in IPMiner study [107].

### Evaluation metrics

To assess the performance of computational predictors, selection of evaluation metrics is an extremely important task to draw definite conclusions. To evaluate the integrity of interaction prediction methodologies, many evaluation metrics have been proposed which mainly compare actual class with predicted class. Here, we assess the performance of the proposed BoT-Net methodology in terms of 7 extensively utilized [33, 82, 104] evaluation metrics including accuracy (ACC), precision (Pre), specificity (SP), sensitivity (SN), F1-score, matthews correlation coefficient (MCC), and area under receiver operating characteristics (AUC-ROC). A short description of these evaluation metrics along with mathematical expression is given below:

17 $$\begin{aligned} f(x)={\left\{ \begin{array}{ll} \text {ACC}= \frac{(O_{-}^{+}+ (O_{+}^{-})}{(O^{+}+ O^{-})} \\ \text {SPE}= \frac{(O_{+}^{-})}{(O_{+}^{-})+ (F_{-}^{+}) } \\ \text {PRE}= \frac{(O_{-}^{+})}{(O^{+})} \\ \text {SEN}= \frac{(O_{-}^{+})}{(O_{-}^{+})+ (F_{+}^{-})} \\ \text {MCC}= \frac{\frac{O_{-}^{+}}{O^{+}}+ \frac{O_{+}^{-}}{O^{-}}}{\sqrt{(1+O_{+}^{-}-O_{-}^{+}/O^{+}) (1+O_{-}^{+}- O_{+}^{-}/O^{-}})} \\ \text {F1-score}= 2 * \frac{[Pre*SN]}{ [Pre+SN]} \end{array}\right. } \end{aligned}$$

In Eq. 17, O$^{+}$ indicates the bunch of true and false positives, while O$^{-}$ refers to the bunch of true and false negatives. The proportion of interactive lncRNA–miRNA sequences that are correctly classified as interactive are expressed using $O^+_-$ and the proportion of non-interactive lncRNA–miRNA sequences that are accurately identified as non-interactive are expressed using $O^-_+$. lncRNA–miRNA sequences incorrectly classified as interactive (False positives) are indicated by $F_{-}^{+}$ and lncRNA–miRNA sequences inaccurately classified as non-interactive (False Negatives) are referred as $F_{+}^{-}$.

Accuracy (ACC) computes the ratio of accurately predicted interactive and non-interactive instances with total predictions made by the model. However, in the case of dataset having imbalance classes, it fails to reveal the correct performance of the model. Specificity and sensitivity compute true negative and true positive rate in turn, whereby, precision estimates up to what extent positive predictions are fully correct. F1 score computes the harmonic mean between precision and sensitivity. Evaluation criterion such as precision, sensitivity, and F1 score can be classified as asymmetric as they fully ignore true negatives and their calculation is largely affected by positive class size. On the other hand, MCC, considers all four true negatives, true positives, false negatives, and false positives, into account to compute the performance of the model. A high value of MCC indicates that the model is efficiently discriminating different corpus classes even when a particular class is under or over represented in the corpus.

The evaluation measures discussed primarily assess the performance of the classifier by comparing actual and predicted classes. However, receiver operating characteristic curve (ROC) is computed by making use of actual classes and predicted probabilities which implies the need to find optimal threshold for the hand on problem. Also, few previously discussed evaluation measures such as accuracy correctly reveal the performance of classifier when the classification problem is highly balanced indicating its biasedness towards the majority class. Whereas, area under receiver operating characteristics curve (AU-ROC) is the most widely used evaluation metric which is neither biased towards majority class nor minority class as it measures the classifier performance using different thresholds.

### Experimental setup

The proposed BoT-Net methodology is implemented using Pytorch. Deep exploratory analysis of the benchmark lncRNA–miRNA interaction dataset indicates that the length of miRNA sequences fluctuates from 17 to 25 residues and the length of lncRNA sequence fluctuates from 213 to 22,765 with an average length of 1424 residues. To determine up to what number of residues can effectively capture the discriminative essence of very flexible lncRNA sequences, experimentation is performed by considering as minimum residues as possible (e.g 5 which is around 3% of minimum lncRNA sequence length in benchmark dataset) and keeps increasing this number up to 25% of average lncRNA sequence length using the step size of 5 residues. In benchmark lncRNA–protein interaction prediction dataset, lncRNA sequence length varies from 15 to 1504 and protein sequence length varies from 5 to 1733, indicating that both lncRNA and protein sequences have high length variability. In our experimentation, we only report the performance values produced by BoT-Net by varying residues from 5 to 50 using the step size of 5 residues for lncRNA–miRNA and lncRNA–protein interaction prediction tasks. Unlike lncRNA–miRNA interaction prediction task where we generate sub-sequences of only lncRNAs and take full length of miRNAs due to high length variability of lncRNAs and low length variability of miRNAs. In lncRNA–protein interaction prediction task, both lncRNA and protein sequences have length variability. Hence, we generate sub-sequences of both lncRNA and protein sequences.

To perform an unbiased performance comparison of the proposed BoT-Net methodology with existing lncRNA–miRNA [33, 82, 104] and lncRNA–protein interaction predictors [105], 5-fold cross-validation is performed. Considering, the performance of deep learning models is largely influenced by different values of various hyperparameters such as k-mers, residue embedding dimensions, embedding and standard dropout, learning rate, weight decay, batch size, etc. From the training set, we use 10% sequences as the validation set to find the optimal values of the most influential hyperparameters for lncRNA–miRNA and lncRNA–protein interaction prediction tasks using grid search [41, 59]. To ensure reproduceability of the results, Table 1 reports the initial value range for different hyperparameters defined by following the literature [1, 2] and the optimal hyperparameter values found through the grid search for proposed BoT-Net approach for lncRNA–miRNA and lncRNA–protein interaction prediction tasks.

**Table 1:** Different hyperparameter ranges and optimal values used by the proposed BoT-Net method for lncRNA–miRNA interaction prediction and lncRNA–protein interaction prediction tasks

Hyperparameters	Residue range (k-mers)	Stride	Residue embedding dimensions	Embedding vector dropout	Embedding vector weight dropout	LSTM hiddent units	DropConnect rate	Learning rate	Weight decay	Standard dropout	Batch size
lncRNA–miRNA interaction prediction task											
Hyperparameters ranges in grid search	{1,2,3,4,5}	{1,2,3}	{100, 120, 180, 200, 240, 300}	{0.001, 0.002, 0.003, 0.004, 0.005}	2E3436 {0.001, 0.002, 0.003, 0.004, 0.005}	{2⌃4, 2⌃5, 2⌃6, 2⌃7}	{0.001, 0.002, 0.003, 0.004, 0.005}	{0.01, 0.02, 0.03, 0.04, 0.05}	{0.01, 0.02, 0.03, 0.04, 0.05}	{0.1, 0.2, 0.3, 0.4, 0.5}	{8, 16, 32, 64}
Optimal values of hyperparameters	5	1	120	0.004	0.005	2⌃4	0.004	0.01	0.02	0.1	64
lncRNA–protein interaction prediction task											
Hyperparameters ranges in grid search	{1,2,3,4,5}	{1,2,3}	{100, 120, 180, 200, 240, 300}	{0.001, 0.002, 0.003, 0.004, 0.005}	{0.001, 0.002, 0.003, 0.004, 0.005}	{2⌃4, 2⌃5, 2⌃6, 2⌃7}	{0.001, 0.002, 0.003, 0.004, 0.005}	{0.01, 0.02, 0.03, 0.04, 0.05}	{0.01, 0.02, 0.03, 0.04, 0.05}	{0.1, 0.2, 0.3, 0.4, 0.5}	{8, 16, 32, 64}
Optimal values of hyperparameters	1	1	120	0.004	0.005	2⌃4	0.004	0.05	0.01	0.4	64

## Results

This section comprehensively illustrates the performance produced by three trivial sequence fixed-length generation approaches based on standard copy padding and sequence truncation paradigms. Furthermore, it compares the performance of three different settings which select a different number of residues solely from the start (only start), end (only end), and start-end regions of lncRNA sequences with an aim to find and retain the most informative residue distribution-based lncRNA sub-sequences with respect to lncRNA–miRNA interaction prediction. It compares the performance of the proposed BoT-Net approach with existing sequence-based lncRNA–miRNA interaction prediction approaches. Finally, it performs a case study analysis based on the task of lncRNA–protein interaction prediction to validate the effectiveness and generalizeability of proposed sub-sequences-based BoT-Net approach.

**Fig. 3:**
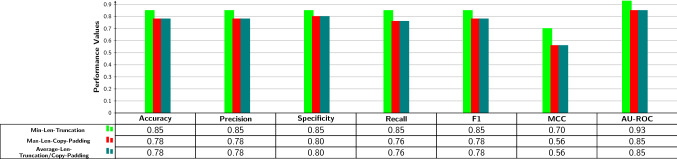
Performance comparison of traditional fixed-size sequence representation generation schemes based on minimum (min-len-truncation), maximum (max-len-copy-padding), and average (average-len-truncation/copy-padding) length of sequences in terms of seven different evaluation metrics

Figure 3 illustrates the performance produced by 3 fixed-length sequence generation approaches for the task of lncRNA–miRNA interaction prediction.

As indicated by the bar graph shown in Fig. 3, among all three approaches, min-len-truncation approach which maps the miRNA and lncRNA sequences to their respective minimum length and truncates additional residues marks the best performance in seven different evaluation metrics. It outperforms the performance of the other two approaches by 5% in five different evaluation metrics, 8% in terms of AU-ROC, and 14% in terms of MCC. Whereas, other two approaches namely max-len-copy-padding and average-len-truncation/copy-padding, which map miRNA and lncRNA sequences to their respective maximum and average length mark exactly the same performance across all seven evaluation metrics.

**Fig. 4:**
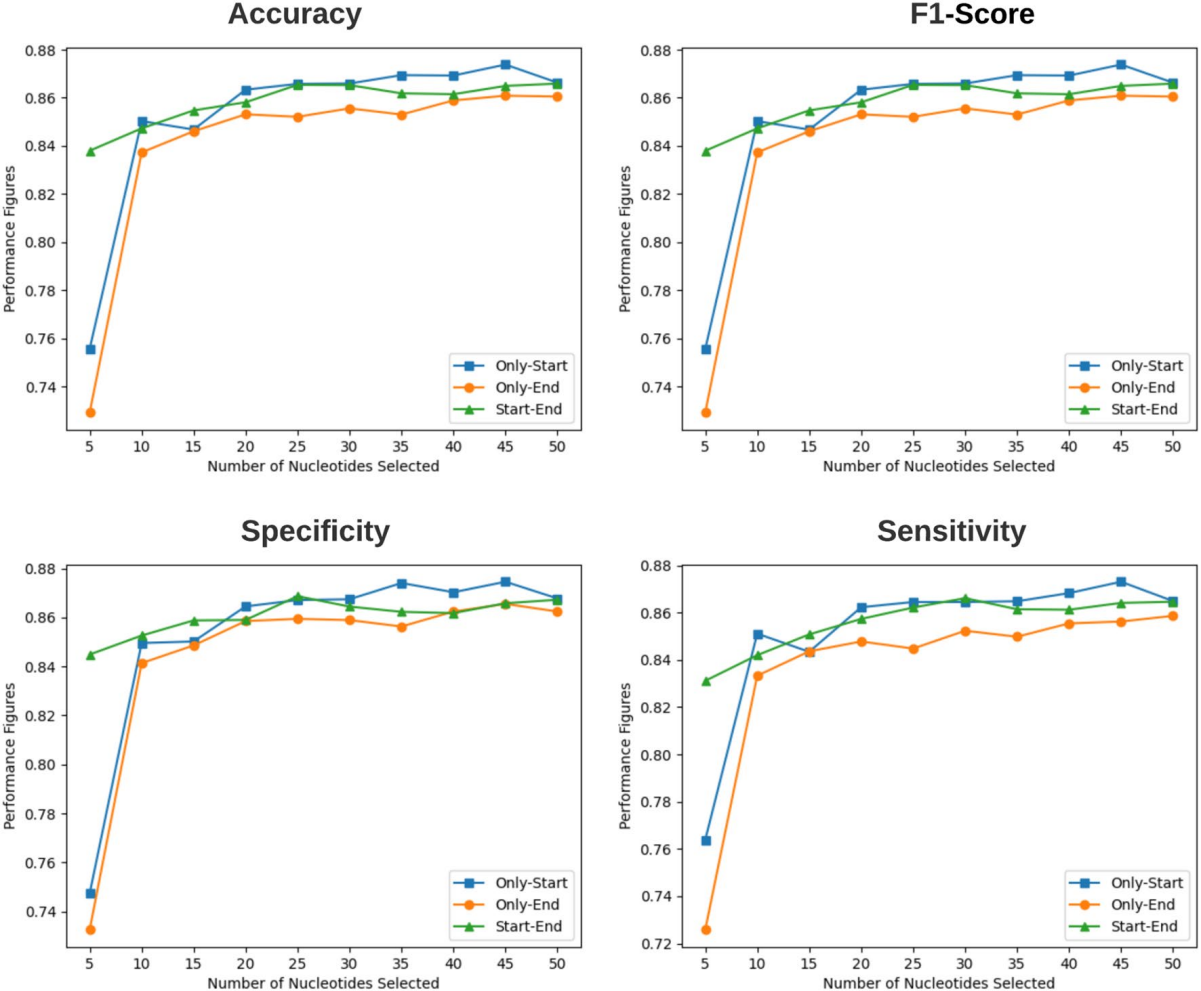
Performance produced by proposed bot-net methodology using three different most informative and discriminative residue distribution-based sub-sequence generation settings in terms of four distinct evaluation metrics. Setting 1–3 selects different number of residues solely from start, end, and start-end respectively

Furthermore, performance figures produced by BoT-Net approach using three different sub-sequence-based settings are illustrated using accuracy, F1-score, sensitivity, and specificity line graphs, shown in Fig. 4.

Analysis of the accuracy and F1-score corresponding pictorial representations (Fig. 4) indicates that the performance of BoT-Net starts in low figures with 5 residues selected by only-start setting or only-end setting. Using 10 residues, the BoT-Net performance is rocketed for both settings. With the further increase in residues, in only-start setting, BoT-Net performance fluctuates by the figure of 1% across 2 thresholds before stabilizing until 30 residues. Then, it increases further to achieve peak performance figures of 87% in both evaluation metrics. Whereas, in only-end setting, BoT-Net performance gradually increases until 20 residues, slightly fluctuates afterward until 40 residues before leveling off and ending at 85.5% in terms of accuracy and F1-score respectively. Unlike only-start and only-end setting, over start-end setting, the BoT-Net performance starts with decent figures and almost gradually increases until 25 residues, but gradually declines thereafter until 40 residues before leveling at 86% in terms of accuracy and F1-score (Fig. 4).

Taking the sensitivity of BoT-Net into account, Fig. 4 reveals that BoT-Net marks a similar performance trend with both only-start and only-end setting, however, it achieves better sensitivity figures across most test points using only-start setting. In start-end setting, BoT-Net performance gradually increases until 30 residues before gradually declining and leveling off. In terms of specificity, an identical performance pattern is evident in all settings except the start-end setting in Fig. 4. In start-end setting, the performance of BoT-Net gradually increases to 15 residues before fluctuating moderately at other residue thresholds. Overall, only-start setting once again marks better performance across most test points.

**Fig. 5:**
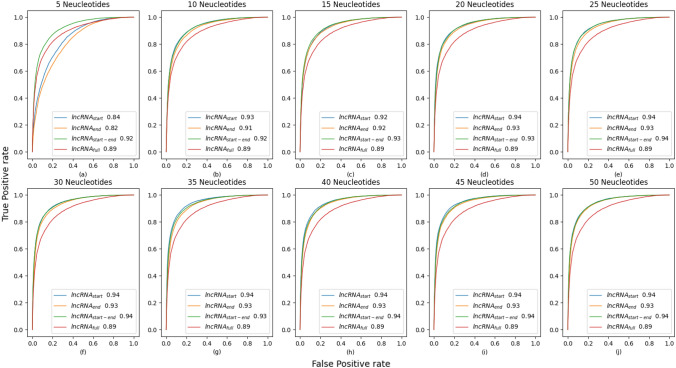
AU-ROC produced by proposed BoT-Net Methodology using full LncRNA sequence and three different most informative residue distribution-based sub-sequence generation settings without slicing mirma sequences. Setting 1–3 selects different number of residues solely from start, end, and start-end, respectively

Furthermore, the performance potential of full length lncRNA–miRNA sequence pairs is compared with three different lncRNA–miRNA sub-sequence-based settings in terms of area under receiver operating characteristics (AU-ROC).

It is evident from Fig. 5 that with the influx of residues, the performance of all three sub-sequence-based settings gets improved. With just ten residues, the performance of all three experimental settings jumps over 90% as compared to 89% achieved using the complete lncRNA sequences. Among all three sub-sequence settings, only-start setting outperforms other settings across nine test iterations out of ten test iterations. As indicated by other evaluation metrics, here again, start-end setting outshines other settings using five residues and marks the second best performance of 94% in most test iterations. Whereas, the performance of the only-end setting first jumps from 82% to 91% in the first two iterations and then increases by the figure of 1% until 20 residues before leveling off at 93%. Furthermore, the performance of all 3 sub-sequence-based settings is affected until 25 residues, however afterward, it remains the same across all remaining test iterations.

To summarize, the performance potential of different regions of lncRNA sequences is definite. BoT-Net marks best performance with the only-start setting followed by the only-end setting and the start-end setting.

To prove the integrity of the proposed BoT-Net methodology, the performance figures produced by the proposed BoT-Net methodology are compared with existing computational predictors in Table 2 in terms of six distinct evaluation metrics.

**Table 2:** Performance Comparison of Proposed BoT-Net methodology with Existing Sequence-based lncRNA–miRNA Interaction Prediction Approaches in terms of 6 Different Evaluation Metrics: Performance Figures of Existing Predictors are taken from Table 2 of State-of-the-art Paper (LncMirNet) [82]

Approach	Accuracy	Specificity	Sensitivity	F1-score	MCC	AU-ROC
GEEL [104]	0.8220	0.8401	0.8040	0.8187	0.6445	0.8982
PmliPred [33]	0.7959	0.7118	0.8800	0.8117	0.6004	0.9030
BiLSTM [82]	0.7145	0.6263	0.8027	0.7239	0.4359	0.7876
SEAL [82]	0.7874	0.8097	0.7650	0.7825	0.5754	0.8658
SVD [82]	0.6571	0.6594	0.6548	0.6595	0.3142	0.7156
Katz [82]	0.5964	0.5961	0.5969	0.5953	0.1930	0.6459
LncMirNet [82]	0.8534	0.7910	0.9158	0.8620	0.7124	0.9381
Proposed BoT-Net	0.8738	0.8746	0.8731	0.8738	0.7477	0.9449

As indicated by the Table 2, proposed purely sequence information-based BoT-Net methodology significantly outshines the performance of existing sequence-based predictors across almost all evaluation metrics. More specifically, it outperforms state-of-the-art LncMir-Net performance by the figure of 2% in terms of accuracy, 8% in terms of specificity, 1% in terms of F1-score, and 4% in terms of MCC. A significant fluctuation in LncMir-Net specificity and sensitivity performance figures proves that state-of-the-art predictor is biased towards type I or type II error. Whereas, BoT-Net methodology performance is stable and superior than all predictors primarily due to two reasons. On one hand, BoT-Net utilizes the most informative residue-based sub-sequences of LncRNA sequences. On the other hand, BoT-Net integrates multiple deep learning strategies at a different level of the neural network to enhance the generalizability of the model. Due to these unique properties, BoT-Net manages to obtain consistent performance across different evaluation metrics and overall a decent increment than state-of-the-art lncRNA–miRNA interaction prediction performance.

### A case study: objective evaluation of the proposed BoT-Net methodology

We have seen in previous sections that the novel paradigm of retaining only most informative residues distribution-based sub-sequences helps the proposed BoT-Net approach to most efficiently characterize highly flexible lncRNA sequences for the task of lncRNA–miRNA interaction prediction. To validate the versatility, generalizeability, and practical significance of sub-sequence-based BoT-Net approach, we consider a similar task namely lncRNA–protein interaction prediction to perform a case study analysis. Using five-fold cross-validation on a benchmark RP1369 dataset [105], we first evaluate the performance potential of the proposed BoT-Net methodology using three traditional fixed length sequence generation settings. Then, we illustrate the performance potential of three different sub-sequence-based settings. Finally, we compare the performance of proposed BoT-Net approach with state-of-the-art lncRNA–protein interaction predictor [105].

**Fig. 6:**
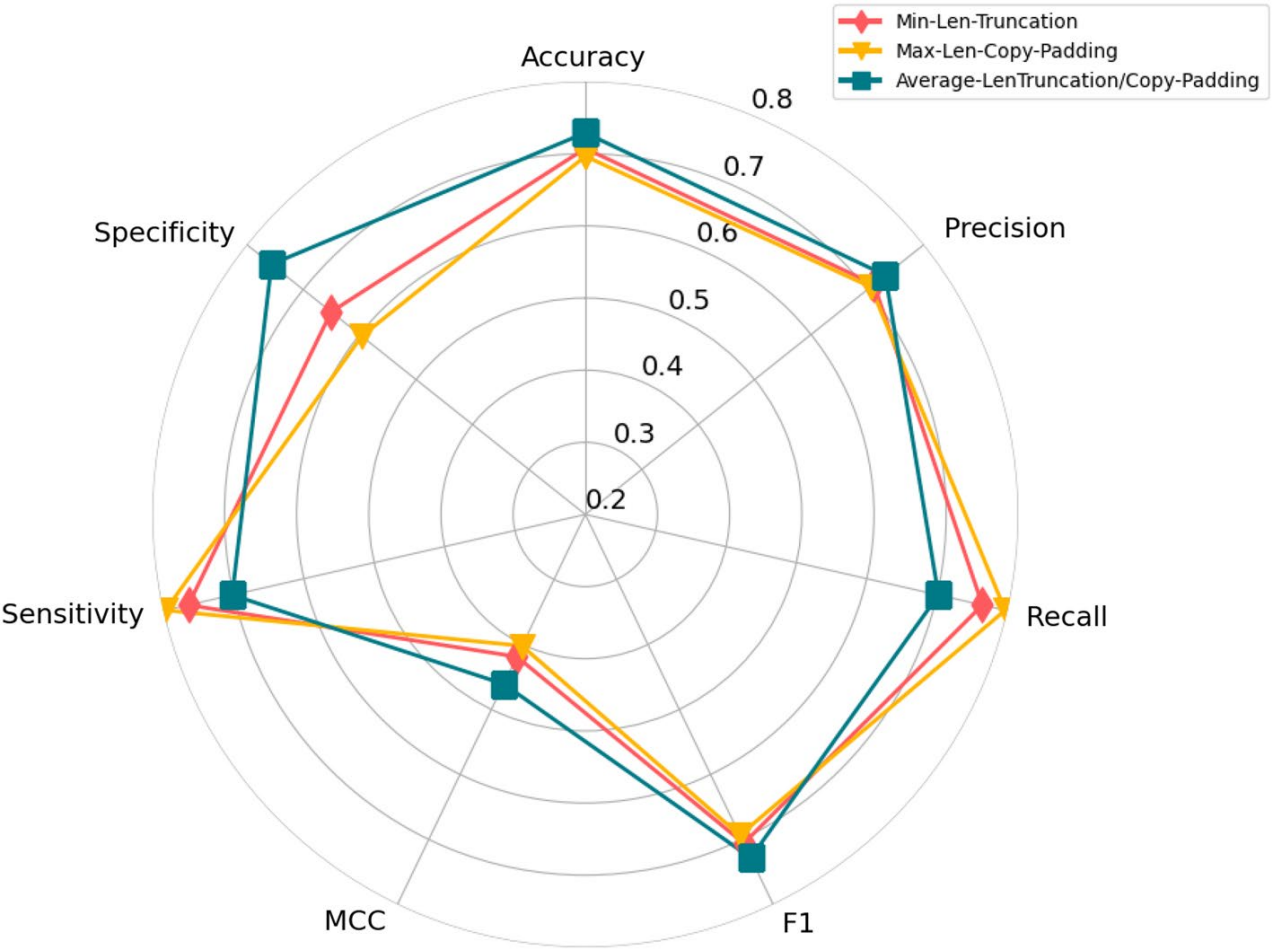
Performance radar graph produced by proposed BoT-Net methodology on a benchmark dataset RP1369 for the task of lncRNA–protein interaction prediction using traditional fixed-size sequence representation generation schemes based on minimum (min-len-truncation), maximum (max-len-copy-padding), and average (average-len-truncation/copy-padding) length of sequences in terms of 7 different evaluation metrics

The radar graph in Fig. 6 reports the performance produced by the proposed BoT-Net methodology using the min-len-truncation, max-len-copy-padding, and average-len-truncation/copy padding settings in terms of seven different evaluation metrics.

As shown by the Fig. 6, among all 3 traditional sequence fixed length generation settings, average-len-truncation/copy padding setting which maps all lncRNA and protein sequences to respective average length achieve better performance across most evaluation metrics, achieving the accuracy as well as F1 score of top accuracy of 72%. The second best performance is attributed to both min-len-truncation and max-len-copy-padding settings as both settings mark almost similar performance across most evaluation metrics.

**Fig. 7:**
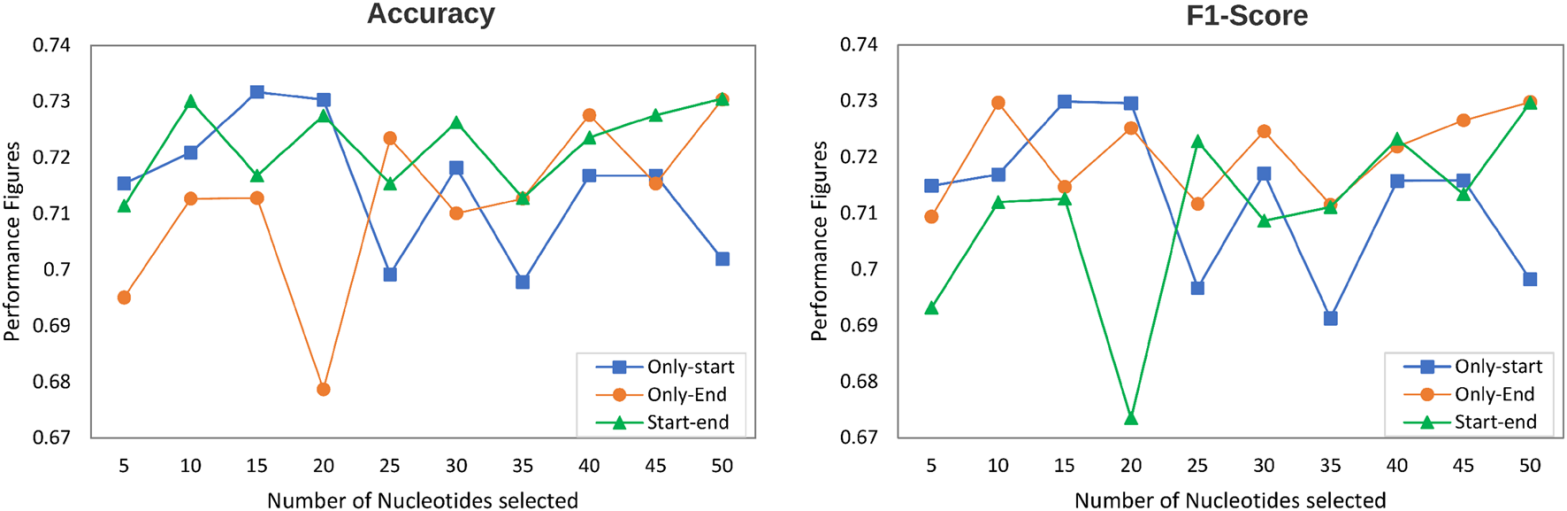
Performance Produced by Proposed BoT-Net Methodology on a Benchmark Dataset RP1369 [105] for lncRNA–protein Interaction Prediction [105] Task using three Different most Informative and Discriminative Residue Distribution-based Sub-Sequence Generation Settings in terms of four Distinct Evaluation Metrics. Only-start, Only-end, and Start-End Settings Select a Different Number of Residues Solely from Starting, Ending, and Starting-Ending regions of lncRNA and Protein Sequences, respectively

Figure 7 illustrates the accuracy and F1 score produced by proposed BoT-Net methodology using merely *k* residues from only start region, only end region, and start-end regions of lncRNA and protein sequences.

It is evident from the Fig. 7 that the performance of BoT-Net increases with the increase in residues to 15 number of residues and achieve the peak accuracy as well as F1 score of 73% using only start region-based sub-sequences. Afterwards, BoT-Net performance fluctuates across different number of residues. Using only end region-based sub-sequences and start-end region-based sub-sequences, BoT-Net performance fluctuates across most thresholds of resides, achieving the peak performance figures of and 72.7% using 50 residues.

**Fig. 8:**
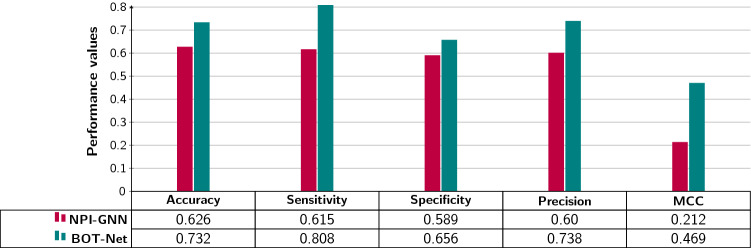
Performance comparison of proposed bot-net methodology with existing sequence-based lncRNA–protein interaction prediction approach namely NPI-GNN [105] using a benchmark dataset RP1369 [105] in terms of five different evaluation metrics: the NPI-GNN performance figures for a benchmark dataset RP1369 are taken from Table 3 present in the State-of-the-art Paper [105]

Bar graph (Fig. 8) compares the performance of proposed BoT-Net methodology with state-of-the-art graph neural network-based lncRNA–protein interaction prediction approach namely NPI-GNN [105].

As shown by the Fig. 8, proposed BoT-Net approach significantly outperforms NPI-GNN approach across all five evaluation metrics, accuracy by 10%, sensitivity by 19%, specificity by 6%, precision by 14%, and MCC by 26%. This proves BoT-Net promising generalizeability across multiple datasets and practical significance for similar interaction prediction tasks.

## Discussion

With the influx of RNA sequencing technologies, several novel miRNAs and lncRNAs have been identified in a variety of species [68]. To explore their core functionality and key roles in diverse biological and pathological processes, determining the interaction between them is indispensable. In the race to develop more robust and generalized lncRNA–miRNA interaction predictors, predominant computational approaches [6, 14, 19, 20, 43, 43, 74, 78, 90, 91, 93, 96, 97, 100–103] rely on some kind of known intrinsic information (eg expression profile similarity network, functional similarity) to determine the interaction between lncRNAs and miRNAs. The more comprehensive the information, the better the model identifies potential lncRNA–miRNA interactions. Likewise, researchers have explored protein secondary, tertiary or quarternary structural information [108], locality of spatial structures on protein binding-motif regions [108], compatibility levels of different protein regions [108], etc, along with intrinsic information of lncRNAs for accurate lncRNA–protein interaction prediction task. Major overhead of such approaches is the need of constant accumulation of intrinsic information in order to prove effective for novel lncRNA and miRNA sequences or protein sequences which hinders the establishment of an efficient large-scale interaction prediction landscape for multiple species.

Evidently, sequence information-based computational approaches [33, 82, 104, 105] are more scalable and efficient as they do not require expensive expression profile similarity networks. However, existing computational approaches mark confined performance primarily because of their inability to handle high length variability of only lncRNA sequences or both lncRNA and protein sequences. In this regard, proposed BoT-Net novel paradigm of finding and retaining only most informative higher order residue-based lncRNA sub-sequences optimize the process of generating fixed length lncRNA–miRNA sequence pairs and lncRNA–protein sequence pairs for primary lncRNA–miRNA interaction prediction task and case study lncRNA–protein interaction prediction task. Furthermore, instead of leveraging deeper or wider neural networks to effective capture the non-linearity of sequences, BoT-Net leverages a precise deep learning model which is regularized and optimized by making best use of diverse neural tricks. Unlike other neural network, BoT-Net is an extremely powerful model which can be easily trained over hundreds and thousands of lncRNA–miRNA or lncRNA–protein sequence pairs in no time even using a trivial multi-core central processing unit due to having least number of trainable parameters. In summary, a significant increase in state-of-the-art performance of both lncRNA–miRNA and lncRNA–protein interaction prediction tasks over 2 different benchmark datasets in terms of different evaluation metrics prove the versatility, generalizeability, and practical significance of proposed BoT-Net methodology. Unlike state-of-the-art approaches, the paradigm of retaining only most informative residue distribution-based sub-sequences help the BoT-Net classifier to achieve promising performance across multiple datasets and similar interaction prediction tasks. Although BoT-Net shows great promise for the determination of interaction between different biomolecules. However, it can be anticipated that one of the limitation of proposed BoT-Net methodology is that it may not perform well for interaction prediction when both biomolecules have very short sequences such as small non-coding RNAs.

## An interactive and user-friendly BoT-Net web server

We have deployed BoT-Net as an interactive and user-friendly web server (https://sds_genetic_analysis.opendfki.de/lncmiRNA/) that genomic researchers and practitioners can use to predict lncRNAs interactions with miRNAs or proteins biomolecules merely using raw sequences. This web server can also be used to validate experimentally detected lncRNA–miRNA and lncRNA–protein interactions by providing lncRNA and miRNA or protein sequence pairs. Furthermore, it can be used to train and optimize the proposed BoT-Net methodology from scratch for lncRNA and miRNA or protein sequence pairs and perform prediction on test lncRNA and miRNA or protein sequence pairs belonging to existing or new species.

## Conclusion

In this study, we present a novel very lightweight yet robust computational approach for lncRNA–miRNA interaction prediction. It solely utilizes raw sequence information to extract the most informative residue distribution-based lncRNA sub-sequences to generate lncRNA–miRNA pairs and makes the best use of different deep learning strategies to achieve optimal classification performance. Fair performance comparison of proposed BoT-Net with state-of-the-art sequence-based computational lncRNA–miRNA interaction predictor as well as a case study analysis based on lncRNA–protein interaction prediction indicates the efficiency and effectiveness of the proposed methodology for different benchmark datasets and similar interaction prediction tasks. Considering the typical length of other non-coding RNA interactive pairs, we consider that the key findings of this study such as performing classification using sub-sequences of most informative residues will largely supplement the bioinformatics researchers in the development of precise, fast-converging yet robust computational approaches capable to accurately identify interactions between other non-coding RNAs. A compelling future of current work would be to combine sequence information with similarity information to investigate whether the addition of similarity information significantly improves the characterization of lncRNA–miRNA sequences.

## Data Availability

The data as well as results are presented within the manuscript.

## Footnotes

1 https://www.gencodegenes.org/.

2 http://www.mirbase.org.

## References

[1] Le NQK, Yapp EKY, Ho Q-T, Nagasundaram N, Ou Y-Y, Yeh H-Y (2019) iEnhancer-5Step: identifying enhancers using hidden information of DNA sequences via Chou’s 5-step rule and word embedding analytical biochemistry. Elsevier, vol 571, pp 53–61. 10.1016/j.ab.2019.02.017 10.1016/j.ab.2019.02.017 30822398

[2] Asim MN, Ibrahim MA, Malik MI, Dengel A, Ahmed S (2020) Enhancer-dsnet: a supervisedly prepared enriched sequence representation for the identification of enhancers and their strength, pp 38–48. 10.1007/978-3-030-63836-8_4

[3] Asim MN, Malik MI, Zehe C, Trygg J, Dengel A, Ahmed S (2020) A robust and precise convnet for small non-coding rna classification (rpc-snrc). IEEE Access 9:19379–19390. 10.1109/ACCESS.2020.3037642

[4] Ba JL, Kiros JR, Hinton GE (2016) Layer normalization. arXiv:1607.06450

[5] Bian E-B, Xiong Z-G, Li J (2019). New advances of lncrnas in liver fibrosis, with specific focus on lncrna-mirna interactions. J Cell Physiol.

[6] Bouba I, Kang Q, Luan Y-S, Meng J (2019). Predicting mirna-lncrna interactions and recognizing their regulatory roles in stress response of plants. Math Biosci.

[7] Chauhan S, Ahmad S (2020) Enabling full-length evolutionary profiles based deep convolutional neural network for predicting dna-binding proteins from sequence. Proteins Struct Funct Bioinform 88(1):15–30. 10.1002/prot.25763 10.1002/prot.25763 31228283

[8] Chen Z, Zhao P, Li F, Marquez-Lago TT, Leier A, Revote J, Zhu Y, Powell DR, Akutsu T, Webb GI et al (2020) ilearn: an integrated platform and meta-learner for feature engineering, machine-learning analysis and modeling of dna, rna and protein sequence data. Brief Bioinform 21(3):1047–1057. 10.1093/bib/bbz041 10.1093/bib/bbz041 31067315

[9] Cheng Z, Huang K, Wang Y, Liu H, Guan J, Zhou S (2017). Selecting high-quality negative samples for effectively predicting protein-rna interactions. BMC Syst Biol.

[10] Chrysostomou C, Seker H, Aydin N (2011) Effects of windowing and zero-padding on complex resonant recognition model for protein sequence analysis, pp 4955–4958. 10.1109/IEMBS.2011.6091228 10.1109/IEMBS.2011.6091228 22255450

[11] Chuai G, Ma H, Yan J, Chen M, Hong N, Xue D, Zhou C, Zhu C, Chen K, Duan B (2018). Deepcrispr: optimized crispr guide rna design by deep learning. Genome Biol.

[12] Cohn D, Zuk O, Kaplan T (2018) Enhancer identification using transfer and adversarial deep learning of dna sequences. BioRxiv. 10.1101/264200

[13] ElAbd H, Bromberg Y, Hoarfrost A, Lenz T, Franke A, Wendorff M (2020). Amino acid encoding for deep learning applications. BMC Bioinform.

[14] Fan Y, Cui J, Zhu QQ (2020). Heterogeneous graph inference based on similarity network fusion for predicting lncrna-mirna interaction. Rsc Adv.

[15] Frankish A, Diekhans M, Ferreira A-M, Johnson R, Jungreis I, Loveland J, Mudge JM, Sisu C, Wright J, Armstrong J et al (2019) Gencode reference annotation for the human and mouse genomes. Nucl Acids Res 47(D1):D766–D773. 10.1093/nar/gky955 10.1093/nar/gky955 PMC6323946 30357393

[16] Gal Y, Ghahramani Z (2016) A theoretically grounded application of dropout in recurrent neural networks, pp 1019–1027

[17] Garbin C, Zhu X, Marques O (2020) Dropout vs. batch normalization: an empirical study of their impact to deep learning. Multim Tools Appl. 10.1007/s11042-019-08453-9

[18] Guo Y, Li M, Xuemei P, Li G, Guang X, Xiong W, Li J (2010). Pred\_ppi: a server for predicting protein-protein interactions based on sequence data with probability assignment. BMC Res Notes.

[19] Hu P, Huang Y-A, Chan KCC, You Z-H (2018) Discovering an integrated network in heterogeneous data for predicting lncrna-mirna interactions, pp 539–545. 10.1007/978-3-319-95930-6_51

[20] Hu P, Huang Y-A, Chan KCC, You Z-H (2019) Learning multimodal networks from heterogeneous data for prediction of lncrna–mirna interactions. IEEE/ACM Trans Comput Biol Bioinform 17(5):1516–1524. 10.1109/TCBB.2019.2957094 10.1109/TCBB.2019.2957094 31796414

[21] Huang G, Liu Z, Van Der Maaten L, Weinberger KQ (2017) Densely connected convolutional networks, pp 4700–4708. 10.48550/arXiv.1608.06993

[22] Huang J-Z, Chen M, Chen D, Gao X-C, Zhu S, Huang H, Min H, Zhu H, Yan G-R (2017). A peptide encoded by a putative lncrna hoxb-as3 suppresses colon cancer growth. Mol Cell.

[23] Huang Y-A, Chan KCC, You Z-H (2018) Constructing prediction models from expression profiles for large scale lncrna–mirna interaction profiling. Bioinformatics 34(5):812–819. 10.1093/bioinformatics/btx672 10.1093/bioinformatics/btx672 PMC6192210 29069317

[24] Huang Y-A, You Z-H, Gao X, Wong L, Wang L (2015) Using weighted sparse representation model combined with discrete cosine transformation to predict protein-protein interactions from protein sequence. BioMed Res Int. 10.1155/2015/902198 10.1155/2015/902198 PMC4641304 26634213

[25] Huang Z-A, Huang Y-A, You Z-H, Zhu Z, Sun Y (2018). Novel link prediction for large-scale mirna-lncrna interaction network in a bipartite graph. BMC Med Genom.

[26] Huang Z-A, Huang Y, You Z-H, Zhu Z, Chang-Qing Yu, Huang W, Guo J (2019). Predicting lncrna-mirna interaction via graph convolution auto-encoder. Front Genet.

[27] Ioffe S, Szegedy C (2015) Batch normalization: Accelerating deep network training by reducing internal covariate shift, pp 448–456. https://proceedings.mlr.press/v37/ioffe15.html

[28] Iosifidis A, Tefas A, Pitas I (2015). Dropelm: fast neural network regularization with dropout and dropconnect. Neurocomputing.

[29] Jalali S, Bhartiya D, Lalwani MK, Sivasubbu S, Scaria V (2013) Systematic transcriptome wide analysis of lncrna-mirna interactions. PloS One 8(2):e53823. 10.1371/journal.pone.0053823 10.1371/journal.pone.0053823 PMC3566149 23405074

[30] Ji J, Xia K, Tang J, Jiang R (2019). Lncrna in tumorigenesis microenvironment. Curr Bioinform.

[31] Joulin A, Grave E, Bojanowski P, Mikolov T (2016) Bag of tricks for efficient text classification. 10.48550/arXiv.1607.01759

[32] Kallen AN, Zhou X-B, Xu J, Qiao C, Ma J, Yan L, Lu L, Liu C, Yi J-S, Zhang H et al (2013) The imprinted h19 lncrna antagonizes let-7 micrornas. Mol Cell 52(1):101–112. 10.1016/j.molcel.2013.08.027 10.1016/j.molcel.2013.08.027 PMC3843377 24055342

[33] Kang Q, Meng J, Cui J, Luan Y, Chen M (2020). Pmlipred: a method based on hybrid model and fuzzy decision for plant mirna-lncrna interaction prediction. Bioinformatics.

[34] Killoran N, Lee LJ, Delong A, Duvenaud D, Frey BJ (2017) Generating and designing dna with deep generative models. 10.48550/arXiv.1712.06148

[35] Kong M, Zhang Y, Da X, Chen W, Dehmer M (2020). Fctp-wsrc: protein-protein interactions prediction via weighted sparse representation based classification. Front Genet.

[36] Koo PK, Eddy SR (2019) Representation learning of genomic sequence motifs with convolutional neural networks. PLoS Comput Biol 15(12):e1007560. 10.1371/journal.pcbi.1007560 10.1371/journal.pcbi.1007560 PMC6941814 31856220

[37] Kozomara A, Birgaoanu M, Griffiths-Jones S (2019). mirbase: from microrna sequences to function. Nucl Acids Res.

[38] Kuang L, Zhao H, Wang L, Xuan Z, Pei T (2019). A novel approach based on point cut set to predict associations of diseases and lncrnas. Curr Bioinform.

[39] Lanzafame M, Bianco G, Terracciano LM, Ng CKY, Piscuoglio S (2018) The role of long non-coding rnas in hepatocarcinogenesis. Int J Mol Sci 19(3):682. 10.3390/ijms19030682 10.3390/ijms19030682 PMC5877543 29495592

[40] LeCun Y, Bottou L, Orr GB, Müller K-R et al (1998) Neural networks: tricks of the trade. Springer lecture notes in computer sciences, vol 1524, no 5–50, p 6. 10.1007/3-540-49430-8_1

[41] Liashchynskyi P, Liashchynskyi P (2019) Grid search, random search, genetic algorithm: a big comparison for nas. 10.48550/arXiv.1912.06059

[42] Lin H, Jiang M, Liu L, Yang Z, Ma Z, Liu S, Ma Y, Zhang L, Cao X (2019). The long noncoding rna lnczc3h7a promotes a trim25-mediated rig-i antiviral innate immune response. Nat Immunol.

[43] Liu H, Ren G, Chen H, Liu Q, Yang Y, Zhao Q (2020). Predicting lncrna-mirna interactions based on logistic matrix factorization with neighborhood regularized. Knowl Based Syst.

[44] Liu S, Yang Y, Jiang S, Tang N, Tian J, Ponnusamy M, Tariq MA, Lian Z, Xin H, Yu T (2018) Understanding the role of non-coding rna (ncrna) in stent restenosis. Atherosclerosis 272:153–161. 10.1016/j.atherosclerosis.2018.03.036 10.1016/j.atherosclerosis.2018.03.036 29609130

[45] Rio AL, Martin M, Perera-Lluna A, Saidi R (2020) Effect of sequence padding on the performance of deep learning models in archaeal protein functional prediction. Sci Rep 10(1):1–14. 10.1038/s41598-020-71450-8 10.1038/s41598-020-71450-8 PMC7471694 32884053

[46] Loshchilov I, Hutter F (2017) Decoupled weight decay regularization. 10.48550/arXiv.1711.05101

[47] Loshchilov I, Hutter F (2018) Fixing weight decay regularization in adam

[48] Marín RM, Šulc M, Vaníček J (2013) Searching the coding region for microrna targets. Rna 19(4):467–474. 10.1261/rna.035634.112 10.1261/rna.035634.112 PMC3677256 23404894

[49] Martin S, Roe D, Faulon J-L (2005). Predicting protein-protein interactions using signature products. Bioinformatics.

[50] Mendizabal-Ruiz G, Román-Godínez I, Torres-Ramos S, Salido-Ruiz RA, Morales JA (2017). On dna numerical representations for genomic similarity computation. PloS One.

[51] Merity S, Keskar NS, Socher R (2017) Regularizing and optimizing lstm language models. 10.48550/arXiv.1708.02182

[52] Miao Y-R, Liu W, Zhang Q, Guo A-Y (2018). lncrnasnp2: an updated database of functional snps and mutations in human and mouse lncrnas. Nucleic Acids Res.

[53] Min X, Zeng W, Chen S, Chen N, Chen T, Jiang R (2017). Predicting enhancers with deep convolutional neural networks. BMC Bioinform.

[54] Muhammod R, Ahmed S, Farid DM, Shatabda S, Sharma A, Dehzangi A (2019). Pyfeat: a python-based effective feature generation tool for dna, rna and protein sequences. Bioinformatics.

[55] Muppirala UK, Honavar VG, Dobbs D (2011). Predicting rna–protein interactions using only sequence information. BMC Bioinform.

[56] Otter DW, Medina JR, Kalita JK (2020) A survey of the usages of deep learning for natural language processing. In: IEEE transactions on neural networks and learning systems. 10.1109/TNNLS.2020.2979670 10.1109/TNNLS.2020.2979670 32324570

[57] Peng W-X, Koirala P, Mo Y-Y (2017). Lncrna-mediated regulation of cell signaling in cancer. Oncogene.

[58] Sachdev K, Gupta MK (2019) A comprehensive review of feature based methods for drug target interaction prediction. J Biomed Inform 93:103159. 10.1016/j.jbi.2019.103159 10.1016/j.jbi.2019.103159 30926470

[59] Shekar BH, Dagnew G (2019) Grid search-based hyperparameter tuning and classification of microarray cancer data, pp 1–8. 10.1109/ICACCP.2019.8882943

[60] Silver D, Schrittwieser J, Simonyan K, Antonoglou I, Huang A, Guez A, Hubert T, Baker L, Lai M, Bolton A et al (2017) Mastering the game of go without human knowledge. Nature 550(7676):354–359. 10.1038/nature24270 10.1038/nature24270 29052630

[61] Singh J, Shailendra S, Dharam V (2019) Classification of non-coding rna-a review from machine learning perspective. https://tinyurl.com/bd5ekafa

[62] Srivastava N, Hinton G, Krizhevsky A, Sutskever I, Salakhutdinov R (2014). Dropout: a simple way to prevent neural networks from overfitting. J mach Learn Res.

[63] Tang W, Wan S, Yang Z, Teschendorff AE, Zou Q (2018). Tumor origin detection with tissue-specific mirna and dna methylation markers. Bioinformatics.

[64] Tang Y, Jinpeng Wang Yu, Lian CF, Zhang P, Yingfen W, Li X, Xiong F, Li X, Li G (2017). Linking long non-coding rnas and swi/snf complexes to chromatin remodeling in cancer. Mol Cancer.

[65] Umarov R, Kuwahara H, Li Y, Gao X, Solovyev V (2018) Promid: human promoter prediction by deep learning. 10.48550/arXiv.1810.01414

[66] Umarov RK, Solovyev VV (2017) Recognition of prokaryotic and eukaryotic promoters using convolutional deep learning neural networks. PloS One 12(2):e0171410. 10.1371/journal.pone.0171410 10.1371/journal.pone.0171410 PMC5291440 28158264

[67] Veneziano D, Marceca GP, Di Bella S, Nigita G, Distefano R, Croce CM (2019) Investigating mirna–lncrna interactions: computational tools and resources, pp 251–277. 10.1007/978-1-4939-9207-2_14 10.1007/978-1-4939-9207-2_14 30963497

[68] Veneziano D, Marceca GP, Bella SD, Nigita G, Distefano R, Croce CM (2019) Investigating mirna–lncrna interactions: Computational tools and resources, pp 251–277. 10.1007/978-1-4939-9207-2_14 10.1007/978-1-4939-9207-2_14 30963497

[69] Voulodimos A, Doulamis N, Doulamis A, Protopapadakis E (2018) Deep learning for computer vision: a brief review. Comput Intell Neurosci. 10.1155/2018/7068349 10.1155/2018/7068349 PMC5816885 29487619

[70] Wan L, Zeiler M, Zhang S, Cun YL, Fergus R (2013) Regularization of neural networks using dropconnect, pp 1058–1066. https://proceedings.mlr.press/v28/wan13.html

[71] Wang D, Zhang Z, Jiang Y, Mao Z, Wang D, Lin H, Dong X (2021) Dm3loc: multi-label mrna subcellular localization prediction and analysis based on multi-head self-attention mechanism. Nucleic Acids Res. 10.1093/nar/gkab016 10.1093/nar/gkab016 PMC8096227 33503258

[72] Wang J, Yang Y, Ma Y, Wang F, Xue A, Zhu J, Yang H, Chen Q, Chen M, Ye L (2020). Potential regulatory role of lncrna-mirna-mrna axis in osteosarcoma. Biomed Pharmacother.

[73] Wang J, Li X, Zhang H (2020) Gnn-pt: enhanced prediction of compound–protein interactions by integrating protein transformer. Quant Methods. 10.48550/arXiv.2009.00805

[74] Wang M-N, You Z-H, Li L-P, Wong L, Chen Z-H, Gan C-Z (2020). Gnmflmi: graph regularized nonnegative matrix factorization for predicting lncrna-mirna interactions. IEEE Access.

[75] Wang W, Guan X, Khan MT, Xiong Y, Wei D-Q (2020) Lmi-dforest: a deep forest model towards the prediction of lncrna-mirna interactions. Comput Biol Chem. 10.1016/j.compbiolchem.2020.107406 10.1016/j.compbiolchem.2020.107406 33120126

[76] Wang Y, Chen X, Liu Z-P, Huang Q, Wang Y, Derong X, Zhang X-S, Chen R, Chen L (2013). De novo prediction of rna–protein interactions from sequence information. Mol BioSyst.

[77] Wong L, Huang Y-A, You Z-H, Chen Z-H, Cao M-Y (2020). Lnrlmi: linear neighbour representation for predicting lncrna-mirna interactions. J Cell Mol Med.

[78] Wong L, Huang Y-A, You Z-H, Chen Z-H, Cao M-Y (2020). Lnrlmi: linear neighbour representation for predicting lncrna-mirna interactions. J Cell Mol Med.

[79] Xie W, Luo J, Pan C, Liu Y (2020) Sg-lstm-frame: a computational frame using sequence and geometrical information via lstm to predict mirna-gene associations. Brief Bioinform. 10.1093/bib/bbaa022 10.1093/bib/bbaa022 32181478

[80] Yang B, Liu F, Ren C, Ouyang Z, Xie Z, Bo X, Shu W (2017). Biren: predicting enhancers with a deep-learning-based model using the dna sequence alone. Bioinformatics.

[81] Yang Q, Jing W, Zhao J, Tianyi X, Han P, Song X (2020). The expression profiles of lncrnas and their regulatory network during smek1/2 knockout mouse neural stem cells differentiation. Curr Bioinform.

[82] Yang S, Yan Wang Yu, Lin DS, He K, Huang L (2020). Lncmirnet: predicting lncrna-mirna interaction based on deep learning of ribonucleic acid sequences. Molecules.

[83] Yao R-W, Wang Y, Chen L-L (2019). Cellular functions of long noncoding rnas. Nat Cell Biol.

[84] Yelmen B, Decelle A, Ongaro L, Marnetto D, Montinaro F, Furtlehner C, Pagani L, Jay F (2019) Creating artificial human genomes using generative models. 10.1101/769091 10.1371/journal.pgen.1009303 PMC7861435 33539374

[85] Yuan Y, Shi Y, Su X, Zou X, Luo Q, Feng DD, Cai W, Han Z-G (2018) Cancer type prediction based on copy number aberration and chromatin 3d structure with convolutional neural networks. BMC Genom 19(6):1–8. 10.1186/s12864-018-4919-z 10.1186/s12864-018-4919-z PMC6101087 30367576

[86] Zhang G, Pian C, Chen Z, Zhang J, Mingmin X, Zhang L, Chen Y (2018). Identification of cancer-related mirna-lncrna biomarkers using a basic mirna-lncrna network. PloS One.

[87] Zhang G, Wang C, Xu B, Grosse R (2018) Three mechanisms of weight decay regularization. 10.48550/arXiv.1810.12281

[88] Zhang H, Saravanan KM, Yang Y, Hossain MT, Li J, Ren X, Pan Y, Wei Y (2020) Deep learning based drug screening for novel coronavirus 2019-ncov. Interdiscip Sci Comput Life Sci. 10.1007/s12539-020-00376-6 10.1007/s12539-020-00376-6 PMC7266118 32488835

[89] Zhang H, Cai K, Wang J, Wang X, Cheng K, Shi F, Jiang L, Zhang Y, Dou J (2014). Mir-7, inhibited indirectly by lincrna hotair, directly inhibits setdb1 and reverses the emt of breast cancer stem cells by downregulating the stat3 pathway. Stem Cells.

[90] Zhang L, Liu T, Chen H, Zhao Q, Liu H (2021). Predicting lncrna–mirna interactions based on interactome network and graphlet interaction. Genomics.

[91] Zhang L, Yang P, Feng H, Zhao Q, Liu H (2021) Using network distance analysis to predict lncrna–mirna interactions. Interdiscip Sci Comput Life Sci. 10.1007/s12539-021-00458-z 10.1007/s12539-021-00458-z 34232474

[92] Zhang L, Guoxian Y, Guo M, Wang J (2018). Predicting protein-protein interactions using high-quality non-interacting pairs. BMC Bioinform.

[93] Zhang P, Meng J, Luan Y, Liu C (2020). Plant mirna-lncrna interaction prediction with the ensemble of cnn and indrnn. Interdiscip Sci Comput Life Sci.

[94] Zhang S-W, Zhang X-X, Fan X-N, Li W-N (2020). Lpi-cnncp: prediction of lncrna-protein interactions by using convolutional neural network with the copy-padding trick. Anal Biochem.

[95] Zhang W, Li Z, Guo W, Yang W, Huang F (2019) A fast linear neighborhood similarity-based network link inference method to predict microrna-disease associations. IEEE/ACM Trans Comput Biol Bioinform. 10.1109/TCBB.2019.2931546 10.1109/TCBB.2019.2931546 31369383

[96] Zhang W, Tang G, Wang S, Chen Y, Zhou S, Li X (2018) Sequence-derived linear neighborhood propagation method for predicting lncrna–mirna interactions, pp 50–55. 10.1109/BIBM.2018.8621184

[97] Zhang W, Tang G, Zhou S, Niu Y (2019). Lncrna-mirna interaction prediction through sequence-derived linear neighborhood propagation method with information combination. BMC Genom.

[98] Zhang X, Wang W, Zhu W, Dong J, Cheng Y, Yin Z, Shen F (2019). Mechanisms and functions of long non-coding rnas at multiple regulatory levels. Int J Mol Sci.

[99] Zhao B-W, Zhang P, You Z-H, Zhou J-R, Li X (2020) Predicting lncrna–mirna interactions via network embedding with integrated structure and attribute information, pp 493–501. 10.1007/978-3-030-60802-6_43

[100] Zhao B-W, Zhang P, You Z-H, Zhou J-R, Li X (2020) Predicting lncrna-mirna interactions via network embedding with integrated structure and attribute information, pp 493–501. 10.1007/978-3-030-60802-6_43

[101] Zhao C, Qiu Y, Zhou S, Liu S, Zhang W, Niu Y (2020). Graph embedding ensemble methods based on the heterogeneous network for lncrna-mirna interaction prediction. BMC Genom.

[102] Zhou S, Yue X, Xu X, Liu S, Zhang W, Niu Y (2019) Lncrna-mirna interaction prediction from the heterogeneous network through graph embedding ensemble learning, pp 622–627. 10.1109/BIBM47256.2019.8983044

[103] Zhou S, Yue X, Xu X, Liu S, Zhang W, Niu Y (2019) Lncrna–mirna interaction prediction from the heterogeneous network through graph embedding ensemble learning, pp 622–627. 10.1109/BIBM47256.2019.8983044

[104] Zhou S, Yue X, Xu X, Liu S, Zhang W, Niu Y (2019) Lncrna-mirna interaction prediction from the heterogeneous network through graph embedding ensemble learning, pp 622–627. 10.1109/BIBM47256.2019.8983044

[105] Shen Z, Luo T, Zhou Y, Yu H, Du P (2021) NPI-GNN: predicting ncRNA–protein interactions with deep graph neural networks. Brief Bioinform 22:bbab051. 10.1093/bib/bbab051 10.1093/bib/bbab051 33822882

[106] Lewis B, Walia R, Terribilini M, Ferguson J, Zheng C, Honavar V, Dobbs D (2010). PRIDB: a protein-RNA interface database. Nucleic Acids Res.

[107] Pan X, Fan Y, Yan J, Shen H (2016). IPMiner: hidden ncRNA-protein interaction sequential pattern mining with stacked autoencoder for accurate computational prediction. BMC Genom.

[108] Liu Z (2020). Predicting lncRNA-protein interactions by machine learning methods: a review. Curr Bioinform.

